# Illumination by short-wavelength light inside the blind spot decreases light detectability

**DOI:** 10.1016/j.isci.2024.110612

**Published:** 2024-07-30

**Authors:** Marina Saito, Kentaro Miyamoto, Ikuya Murakami

**Affiliations:** 1 Department of Psychology, the University of Tokyo, Tokyo 113-0033, Japan; 2 Japan Society for the Promotion of Science, Tokyo 102-0083, Japan; 3 Faculty of Design and Architecture, Nagoya City University, Nagoya 467-8501, Japan; 4 Laboratory for Imagination and Executive Functions, RIKEN Center for Brain Science, Wako 351-0198, Japan

**Keywords:** Sensory neuroscience, Biophysics

## Abstract

Although the optic disk corresponding to the blind spot contains no classical photoreceptors, it contains photopigment melanopsin. To clarify whether melanopsin is involved in light detection, we conducted detection tasks for light stimuli presented in the normal visual field, with and without another illumination inside the blind spot. We found that a blue blind-spot illumination decreased the light detectability on a dark background. This effect was replicable when it was determined immediately after the blind-spot illumination was turned off, suggesting the contribution of a sluggish system rather than scattering. Moreover, the aforementioned effect was not observed when the blind-spot illumination was in red, indicating wavelength specificity in favor of melanopsin’s sensitivity profile. These findings suggest that melanopsin is activated by the blind-spot illumination and thereby interferes with light detection near the absolute threshold. Light detection originating from conventional photoreceptors is modulated by melanopsin-based computation presumably estimating a baseline noise level.

## Highlights

• Light inside the blind spot is unseen but can be received by photopigment melanopsin
• Blue light within the blind spot lowered light sensitivity elsewhere in the darkness
• Sensitivity did not change if instead we used red light to which melanopsin is blind
• Melanopsin may be monitoring the ambient light levels in the detection of weak light

## Graphical abstract

**Figure:**
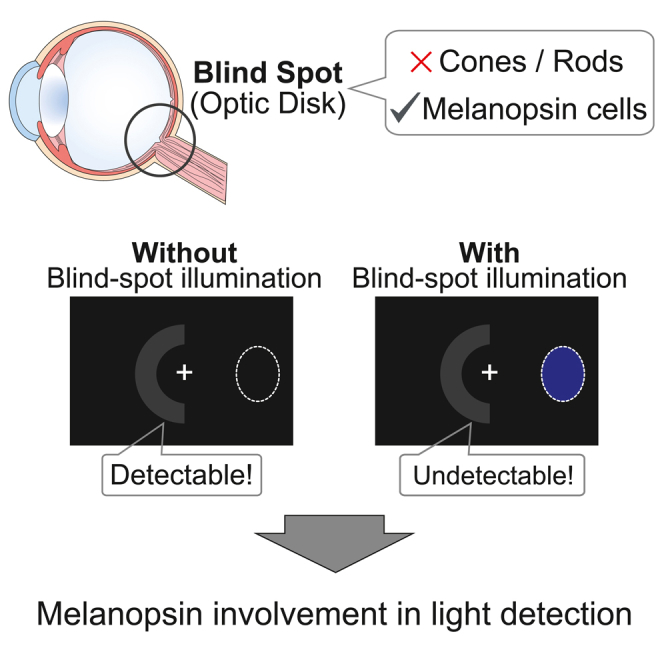

## Introduction

Light detection in darkness is one of the most fundamental and vital visual functions in humans and other animals. Humans can detect subtle signals originating from even a few photons falling on the retina when light is provided in a completely dark environment.^1^ However, the ability to reliably distinguish a real light signal from noise through the orchestration of retinal circuits remains unclear. Although several models have been proposed to explain the absolute threshold, they ignore the potential contribution of melanopsin photopigments in intrinsically photosensitive retinal ganglion cells (ipRGCs), which are distributed ubiquitously throughout the retina to form a distinct neural network. It is difficult to manipulate isolated activation of melanopsin in a dark environment because its absorption spectra overlap with those of classical photoreceptors, rods and cones, which spatially coexist with ipRGCs. However, a convenient method of exclusively stimulating ipRGCs has recently been used along with psychophysical experimentation for healthy human observers.^2^

Individuals with normal vision see a seamless visual world without noticing any missing part within the visual field, even when only one eye is opened. However, there is a part of each monocular retina that cannot receive light inputs as other normal regions do. The optic disk is such a region in the ocular fundus occupied by blood vessels and ganglion cell axons, where no classical photoreceptors exist. Within each monocular visual field, the region corresponding to the optic disk called the blind spot, does not look like a dark hole to the observer, but perceptual filling-in counteracts the lack of visual information by referring to the surrounding visual contexts.^3^ Thus, illumination inside the blind spot is believed to neither elicit any perceptual outcome of its own nor affect any visual functions, such as light detection and brightness judgment. However, a recent physiological study^4^ has revealed that blind-spot illumination enhances the pupillary light reflex (PLR) in response to a light stimulus outside the blind spot. Additionally, another recent psychophysical study^2^ has demonstrated that blind-spot illumination alters brightness perception outside the blind spot. These findings indicate that the blind-spot illumination is received by a mechanism that affects some visual functions. A possible reason can be the scattering of light that occurs through the vitreous body and in the fundus, including the optic disk, which has been refuted in the aforementioned studies through control experiments. As an alternative, contribution from melanopsin has been postulated. Melanopsin is a type of photopigment^5^^,^^6^ with the peak absorbance located at a short wavelength^7^ and a sluggish response time course.^8^ This photopigment is not expressed in classical photoreceptors but in the plasma membranes of the cell bodies of ipRGCs and in their dendrites and axons.^9^ As all the axons of the retinal ganglion cells, including the ipRGCs, converge to the optic disk, they offer an ideal place to elicit simultaneous and exclusive excitation to the ipRGCs within the entire retina by short-wavelength light optimal for melanopsin.^9^

The roles of melanopsin have been examined mainly in non-image-forming vision, such as the systems for circadian rhythm entrainment and light reflex,^10^ and recently, melanopsin has been found to contribute to image-forming vision mediating our conscious experiences such as brightness perception.^11^ The aforementioned consequences of the blind-spot illumination in the form of PLR enhancement and brightness alteration are examples of non-image-forming and image-forming visions, respectively, the latter suggesting that light reception within the blind spot can work as a biological measure of a reference frame with respect to ambient light intensity that is pertinent to brightness perception.

If melanopsin contributes to the PLR and suprathreshold brightness, what influence can it exert on light detection itself? As the suprathreshold color appearance and perithreshold light visibility are fundamentally distinct aspects of the image-forming vision, it is essential to ascertain whether the ipRGCs and retinal light detection mechanism interact in the domain of visibility. In a bright environment, the whole visual field appears brighter when melanopsin is activated.^11^^,^^12^^,^^13^ Thus, it is expected that the blind-spot illumination will change the biological baseline state related to the background luminance level of the entire visual field, thereby affecting light detection on a dark background, presumably in a detrimental manner as observed in the study of suprathreshold brightness appearance.^2^ In addition, melanopsin is also related to the mechanisms of arousal and alerting.^14^^,^^15^ If melanopsin activation enhances arousal or attentional mechanisms, the blind-spot illumination may facilitate light detection. To clarify whether and how melanopsin is involved in our light detectability, we conducted detection tasks for light stimuli presented on a dark background in the normal visual field at luminance near the absolute threshold, with and without another concurrent illumination inside the blind spot.

## Results

### Experiment 1: Detection of a light stimulus near the absolute threshold on a dark background

**Figure 1:**
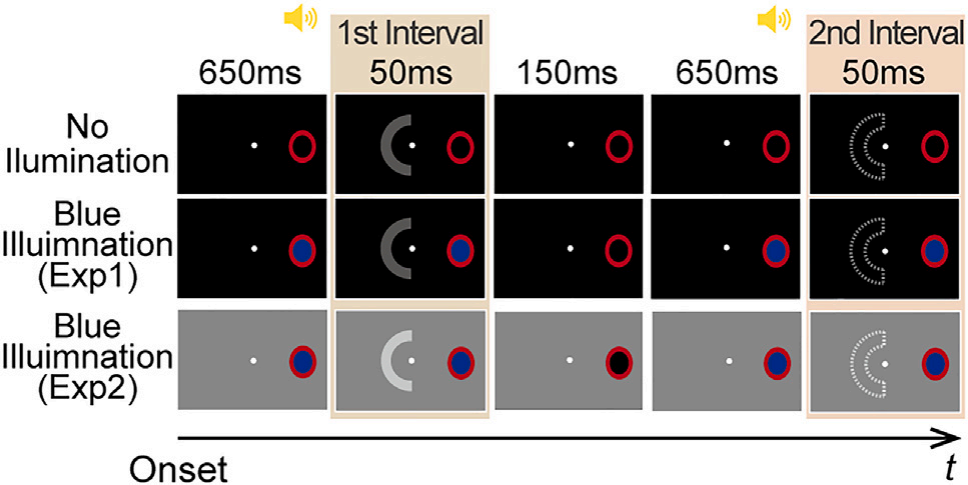
Schematics of the stimuli and procedure The arc was presented during the first interval (indicated by the filled C shape) but not during the second interval (indicated by the C shape with the dashed outline) in these examples, but actually the arc could appear during the first or second interval with 50% probability each. The observers always perceived a filled red oval within the right hemifield because the red annulus induced perceptual filling-in inside the right-eye blind spot, regardless of the presence or absence of the BS stimulus. Each yellow icon of the loudspeaker indicates a beep sound that lasts for 600 ms and is turned off just before each interval.

In experiment 1, we examined the effect of the blind-spot illumination on the detectability of an arc-shaped light stimulus at perithreshold levels of luminance (0.0942–0.106 cd/m^2^; see the STAR Methods section) presented outside the blind spot on a dark background (0.0896 cd/m^2^). The blind spot was illuminated by another short-wavelength light stimulus (“BS stimulus”, 2.71 cd/m^2^). In each trial, the arc was presented in one of two successive “intervals,” each 50 ms long, and the observer was instructed to choose the interval containing the arc (Figure 1). In half of all the trials, the BS stimulus was presented 650 ms before and during both intervals (hereafter referred to as the “blue illumination condition”), and it was absent throughout the trial in the remaining half of the trials (“ni Illumination condition”). The trials were randomized within each block. We analyzed whether the presence or absence of the BS stimulus changed the detectability index (d’) for light detection.

**Figure 2:**
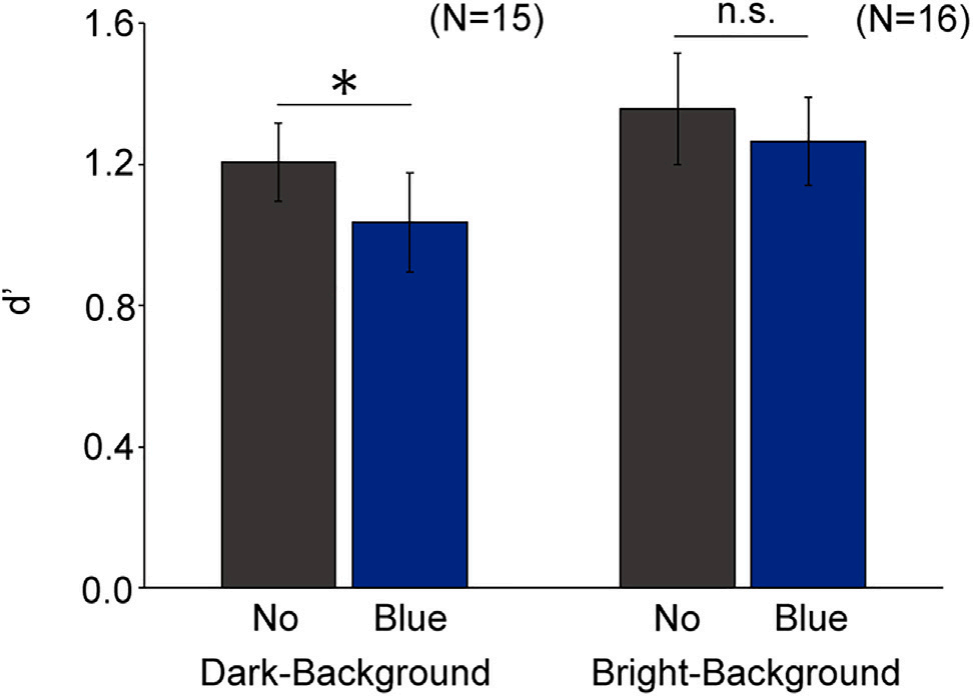
Results of experiments 1 and 2 Light detectability for each condition. The left two bars show the results of experiment 1 (dark-background), whereas the right two bars show the results of experiment 2 (bright-background). The error bars indicate ±1 SEM. ^∗^*p* < 0.05. “n.s.” indicates not significant. “no” indicates the no illumination condition. “blue” indicates the blue illumination condition.

A paired t test revealed that d’ was significantly lower in the blue illumination condition than in the no illumination condition (*t*_14_ = 2.35, *p* = 0.034 < 0.05, the left two bars of Figure 2), suggesting that the BS stimulus was received by some mechanism and affected the visibility outside the blind spot. However, it remains uncertain whether this relationship can be extended to another type of visibility; if the effect of the BS stimulus can be simplified as internal noise, there would be no effect on luminance contrast detection on a bright background, according to the Weber–Fechner law.^16^ Conversely, if the effect operates more like calculation efficiency that stems from attention/arousal, there would still be a similar effect.^17^ Thus, next, we repeated the same experiment on a brighter background.

### Experiment 2: Detection of a light stimulus near the contrast threshold on a bright background

Experiment 2 was identical to experiment 1 (Figure 1), with an identical BS stimulus (2.71 cd/m^2^), except for the luminance of the background outside the blind spot (40.86 cd/m^2^) and the luminance levels of the arc (41.16–43.28 cd/m^2^), the latter of which were optimized to be near approximations of each observer’s contrast threshold. The difference in d’ between the conditions did not reach significance (*t*_15_ = 1.49, *p* = 0.16 > 0.05, the right two bars of Figure 2). The findings are consistent with internal noise, and are also in accord with a previous report on stable contrast sensitivity on a bright background despite blind-spot illumination.^18^ A recent study reported contrast sensitivity increasing 20 min after a 1-min illumination with blue light inside the blind spot, suggesting the involvement of melanopsin in the retinal dopamine.^19^ Unlike such a long-term effect well after the illumination, the present study focuses on the online effect of a brief blind-spot illumination on light detection elsewhere, which might be independent of the retinal dopaminergic modulation.

The results were similar in shape as those in eexperiment 1 but statistically insignificant (*p* = 0.16). It is possible that there was an effect with its size being too weak compared with the power we predetermined according to our prior dataset (see the observers section). It is also possible that the blue illumination, which was identical to experiment 1, was unfairly weak within the framework of experiment 2, because the blind spot was exposed to a bright background whenever the gaze left the fixation point during each inter-trial interval, and because the background was always bright, increasing the persistent activation level of ipRGCs with their somas and dendritic fields constituting receptive fields in the visual field outside the blind spot. Furthermore, we previously reported that blind-spot stimulation reduced the apparent brightness of a suprathreshold stimulus similar to the arc in experiment 2,^2^ and this effect might well be reflected in a reduction in detectability for low contrast stimuli. Future studies will clarify the influence of melanopsin on brightness perception and contrast detection in bright environment, using an improved procedure for ipRGC stimulation and the obtained dataset as a new prior.

### Experiment 3: Detection on a dark background with blind-spot illumination with two colors with a temporal gap

It is to be determined whether our findings in experiment 1 can be explained not by melanopsin but by a scatter of the BS stimulus that may have been received by classical photoreceptors outside the blind spot, instead of melanopsin, thereby reducing the signal-to-noise ratio in the dark background. Thus, an additional experiment was conducted to resolve this issue.

**Figure 3:**
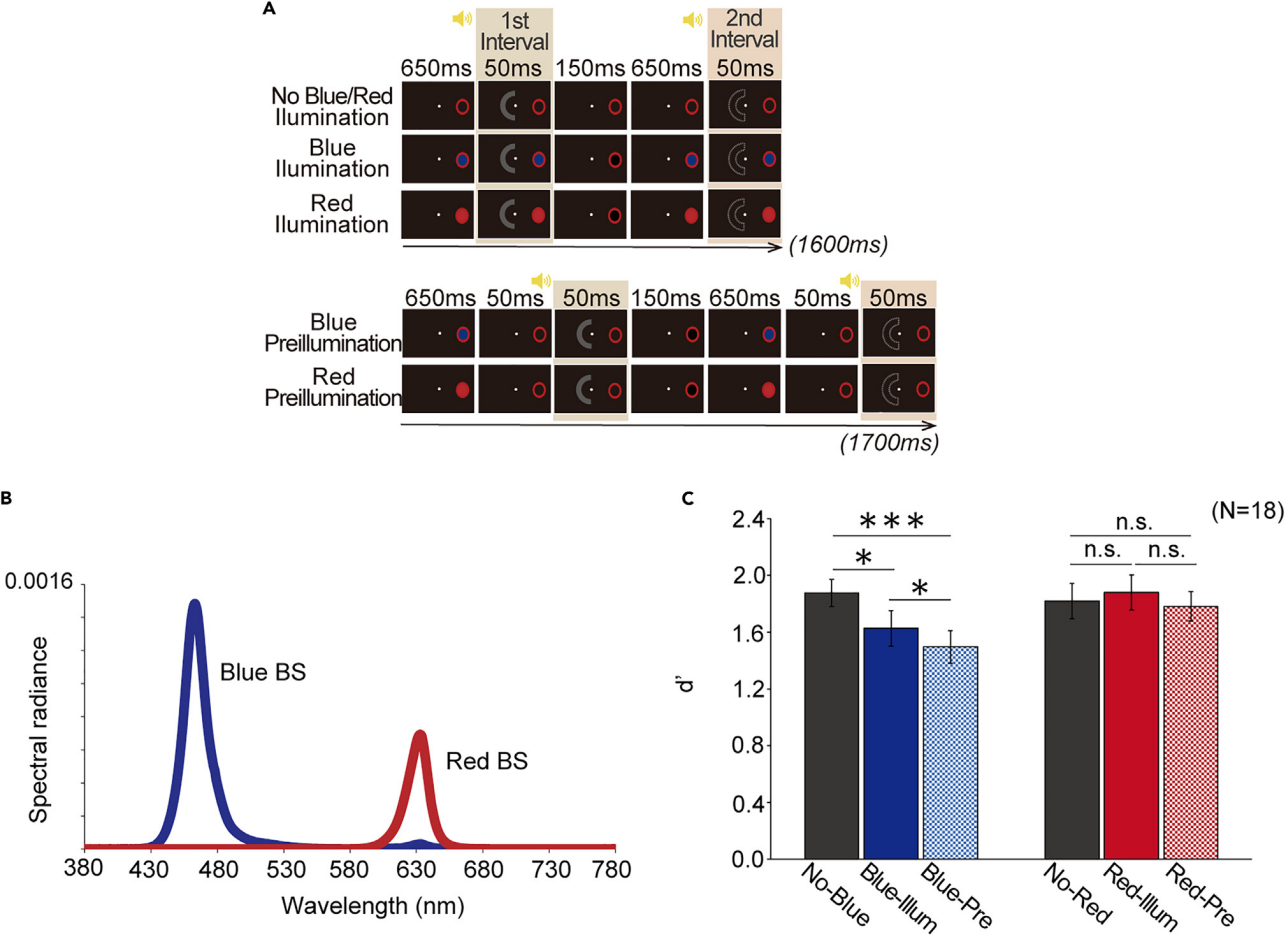
Procedure and results of experiment 3 (A) Schematics of the stimuli and procedure. Each yellow icon of the loudspeaker indicates a beep sound lasting for 600 ms and turned off just before each interval. (B) Spectral components of the blue and red BS stimulus. (C) Light detectability for each condition. The error bars indicate ±1 SEM. ^∗∗∗^*p* < 0.001. ^∗^*p* < 0.05. “n.s.” indicates not significant. “no-blue,” “blue-illum,” “blue-pre,” “no-red,” “red-illum,” and “red-pre” indicates the no blue illumination, blue illumination, blue preillumination, no red illumination, red illumination, and red preillumination conditions, respectively.

ipRGCs retain their visual responses for a short time after stimulus offset.^8^^,^^20^ Additionally, melanopsin-based visual responses are sluggish, and the sensitivity to melanopsin-directed flicker drastically decreases at temporal frequencies >4 Hz, unlike the higher temporal resolutions of classical photoreceptors.^21^ If our results originated from melanopsin, the biological effect of the BS stimulus would linger for some time, even if it was removed from the monitor at the onset of the arc. If, instead, our results originated from scattered light, physical simultaneity would be required to produce this effect. In experiment 3, we introduced a new condition called the “blue preillumination condition” that was identical to the blue illumination condition, except that the BS stimulus was turned off 50 ms before each of the first and second intervals (Figure 3A). The no illumination, blue illumination, and blue preillumination conditions were randomized in each block. With respect to comparisons of d’ across conditions, the melanopsin account predicts that d’ with blue preillumination is lower than d’ with no illumination and that d’ with blue illumination is lower than d’ with no illumination, as observed in experiment 1, which is never predicted by the scatter account and our temporal resolution retained in scotopic vision.^22^

Moreover, we now compared the effect of the blind-spot illumination between short- and long-wavelength bands. As mentioned in the introduction section, the sensitivity peak of melanopsin is found in short-wavelength light, and the sensitivity is lower for long-wavelength light. In experiment 3, we examined whether the same effect would occur if the blind-spot illumination was changed from blue to red of the same luminance (Figure 3B). The melanopsin account predicts that a red BS stimulus would not yield the pattern of results obtained with a blue BS stimulus in experiment 1, whereas the scatter account predicts the same pattern of results as long as the two colors are photometrically equivalent. Thus, we introduced the “red illumination” and “red preillumination” conditions, which were the red counterparts of the aforementioned illumination conditions with blue. In addition, the no illumination condition in experiment 1 was divided into the “no blue illumination” and the “no red illumination” conditions because the blue and red trials were conducted in separate blocks for the experimenter to set up the necessary hardware for each, thereby also preventing any long-term effects of illumination from spilling over across conditions.

A two-way repeated-measures analysis of variance (ANOVA) yielded a significant main effect of color (*F*[1, 17] = 10.99, *p* = 4.09 × 10^−3^ ≪ 0.001) and blind spot condition (*F*[2, 34] = 11.37, *p* = 1.65 × 10^−4^ ≪ 0.001). Their interaction was also significant (*F*[2, 34] = 4.94, *p* = 0.013 < 0.05), indicating that the effect of the blind-spot illumination is different by color (Figure 3C). Using the Tukey-Kramer’s method, we found that the d’ in the no blue illumination condition was significantly greater than that in the blue illumination (*p* = 0.019 < 0.05) and blue preillumination (*p* = 2.73 × 10^−5^ ≪ 0.001) conditions. Interestingly, the d’ in the blue preillumination condition was even significantly smaller than that in the blue illumination condition (*p* = 0.035 < 0.05), suggesting the existence of a sluggish biological mechanism affecting light detection. However, there was no significant difference in d’ between any of the conditions in red (no red illumination vs. red illumination: *p* = 0.83 > 0.05, no red illumination vs. red preillumination: *p* = 0.88 > 0.05, red illumination vs. red preillumination: *p* = 0.34 > 0.05). Color dependencies were also found in pairwise comparisons: d’ was greater in the red illumination than in the blue illumination conditions (*p* = 0.021 < 0.05) and also greater in the red preillumination than in the blue preillumination conditions (*p* = 4.02 × 10^−4^ ≪ 0.001). Finally, there was no significant difference between the two identical but separately executed conditions, namely, no blue illumination and no red illumination conditions (*p* = 0.52 > 0.05). These results support the melanopsin hypothesis.

**Figure 4:**
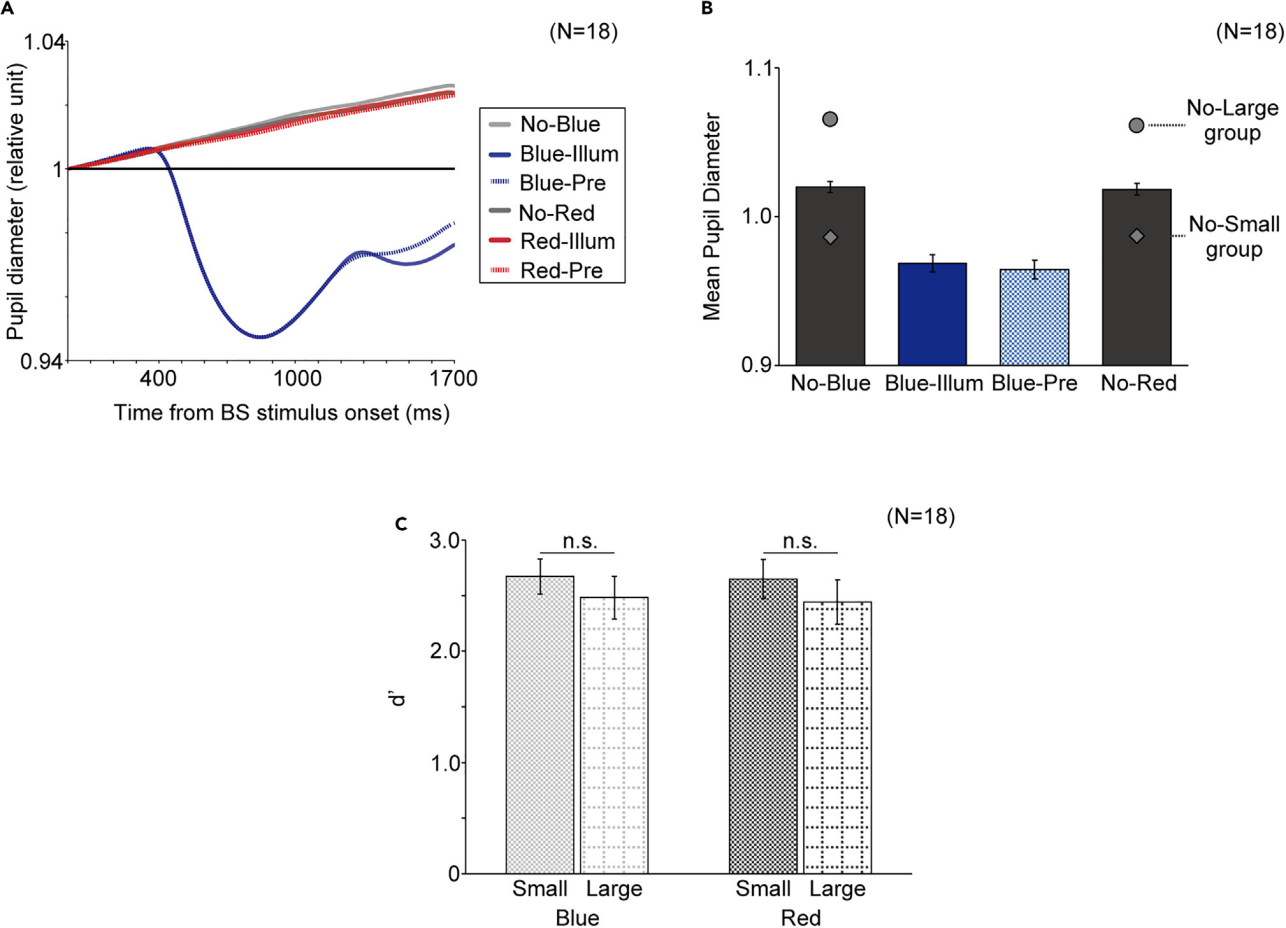
Pupil size in experiment 3 “No-blue,” “blue-illum,” “blue-pre,” “no-red,” “red-illum,” and “red-pre” indicates the no blue illumination, blue illumination, blue preillumination, no red illumination, red illumination, and red preillumination conditions, respectively. (A) Average time course of pupil diameter relative to the baseline. (B) The mean ±1 SEM pupil diameter averaged between the first and second intervals. The circles and the diamonds indicate the mean pupil diameters for the no-(blue/red)-large and no-(blue/red)-small groups, respectively, classified according to the protocol described in the main text. (C) Light detectability for each group. The error bars indicate ±1 SEM. “n.s.” indicates not significant.

Retinal illuminance is proportional to pupil area; therefore, our results might be explained by lower retinal illuminance due to stronger pupil constriction in the blue illumination and blue preillumination conditions than in the no blue illumination condition. In fact, when we examined pupil size during the experiment, it was clear that after the first onset of the BS stimulus, the pupil started to constrict at a latency typical of PLR, with higher shrinkage in the blue illumination and blue preillumination conditions than in any other conditions (Figure 4A), under which the pupil was rather dilated. To obtain an empirical relationship between pupil size and light detectability from the acquired dataset, we averaged the pupil size during the first and second intervals. The difference of interobserver averages in the blue illumination and no blue illumination conditions was 6.90 ± 0.01% (mean ± SEM), and the difference between the blue preillumination and no blue illumination conditions was also 6.90 ± 0.01% (mean ± SEM). There was no statistically significant difference between these two values (paired t test: *t*_17_ = 2.11, *p* = 0.99 > 0.05). If this difference could solely account for the detectability, the same dependence on pupil size would also be found within the trials in the no blue illumination condition, given that pupil size would fluctuate from trial to trial even without the BS stimulus. Thus, we sorted the trials under the no blue illumination and no red illumination conditions in ascending order of pupil size for each observer and extracted a set of trials from the smallest to the first-quartile pupil (“Small” dataset for each color) and another set of trials from the third quartile to the largest pupil (“large” dataset for each color). The differences in pupil size between these two datasets were 7.90% in blue and 7.41% in red, and they were larger than the aforementioned difference in pupil size between with and without the BS stimulus (Figure 4B). Contrary to the retinal illuminance account, there was no significant difference in d’ between these datasets (small vs. large: *F*[1, 17] = 2.53, *p* = 0.13 > 0.05; blue vs. red: *F*[1, 17] = 0.048, *p* = 0.83 > 0.05; interaction: *F*[1, 17] = 0.0044, *p* = 0.95 > 0.05), indicating that pupil size has no effect on the difference in the visibility of the test stimulus (Figure 4C). The same analysis applied to the data of experiment 1 resulted in the same conclusion (see Figure S1).

That the BS stimulus relatively influenced pupil size (Figure 4A) contrasts with the previous study reporting the contrary,^4^ but differences in stimulus conditions leave open the possibility that the BS stimulus alone can cause the PLR, especially when the background is sufficiently dark. However, in experiment 3, we carefully confirmed that none of such biological events could explain our psychophysical results.

## Discussion

Our findings are 4-fold. First, illumination delivered directly inside the blind spot decreased the detectability of another light stimulus presented outside the blind spot (Figure 2). This finding extends a previous report demonstrating that a similar blind-spot illumination reduced the brightness of a stimulus presented outside the blind spot.^2^ Second, the effect was not significant on a bright background (Figure 2), possibly implying that blind-spot illumination is simplified as internal noise in a light sensing mechanism rather than inputs to cognitive controls such as attention and arousal. Third, the effect persisted although the light near the absolute threshold and the blind-spot illumination did not overlap in time. Fourth, the effect of blind-spot illumination disappeared when the stimulus inside the blind spot was red, not blue (Figure 3). Therefore, we conclude that the blind-spot illumination activates some photoreception processes, possibly via the activation of melanopsin expressed in the axons of the ipRGCs passing through the optic disk, and triggers their interactions with the light sensing mechanism, starting from classical photoreceptors residing outside the optic disk of the retina.

### Leakage of the illumination inside the blind spot cannot influence detectability

In this study, a number of careful experimental controls assured that light leakage could not have influenced the detectability of another light stimulus. One, fixation maintenance was constantly monitored by an eye tracker. Two, our BS stimulus was actually smaller than and well inside the mapped blind spot^2^; furthermore, in experiment 3, the smallest size of the BS stimulus in experiment 1 was used for all observers. Three, this oval region was constantly surrounded by a bright red annulus that was equivalent to the BS stimulus in area to cover potential misalignment, aberration, and forward scattering (Figure 1); the red annulus not only provided a constant source of simultaneous masking but also induced perceptual filling-in^23^ with a red apparent surface. Four, the results of the blue preillumination condition in experiment 3 clearly contradicted the effect of scattered light, which must have been dispersed at light speed and dissipated by the time (50 ms after the illumination was turned off) the arc was delivered. In multifocal electroretinogram (ERG) studies, a very weak response to light exposure to the optic disk was observed, suggesting scatter,^24^^,^^25^ but this response disappeared within 50 ms after each light flash.^26^ Five, our effect persisted after this 50-ms time lag, which was deliberately chosen to take into account the activation time course of the classical photoreceptor cells that might have been able to transfer any effect of light scatter to the successively presented arc. Six, the conditions in experiment 3 using red illumination at the same luminance as blue but with virtually no overlap with the absorbance spectra of melanopsin (see Figure 3B and STAR Methods section) were important conditions to test artifacts from misalignment or scatter, and we found no effect on the light detection performance. Lastly, even if one would try to interpret the results of experiment 3 in terms of somewhat stronger scatter for shorter wavelengths,^27^^,^^28^ it is difficult for such scattered light to become sufficiently strong to change light detectability; the luminance of the BS stimulus was only 2.71 cd/m^2^ in the first place, and even if it produced 100% uniform dispersion dropping back to the entire retina, which was more than 1,000 times larger in area than the projected image of the BS stimulus, dispersed light per unit area would be reduced to <0.1% at the reentry to each visual field. In reality, stray light is not uniform throughout the whole visual field, with its intensity dropping systematically with the distance from the light source,^29^ producing retinal reentry that must be too weak to account for the effect under the illumination and preillumination conditions in experiment 3.

### Blind-spot illumination as a model case for photoreception throughout the visual field

As mentioned previously, a blind spot is a special region. It would be improbable in daily life to experience the highly artificial situation we introduced, namely, light exposure exclusively inside the blind spot. However, the fact that the axons of all ganglion cells converge in the optic disk means that our blind-spot illumination mimics the situation in which environmental illumination uniformly and simultaneously activates all of the affected ipRGCs whose receptive fields are widely spread throughout the visual field.^8^ This situation seems to be naturally plausible if we consider that the ipRGCs are designed to receive light information not within a tiny receptive field, but from a large spatial extent, as surmised from their way of arborization and interconnectivity.^8^ In other words, the present study demonstrates that light detection near the absolute threshold is apparently exacerbated when the ipRGCs receive fake global light information, suggesting that the system involving the ipRGCs normally plays a role in monitoring the overall light level of the surrounding environment, updating the prior knowledge about noise, or a baseline for light detection, to maintain detectability while minimizing incorrect responses that may stem from external noise.

### Usefulness of the blind spot in research on melanopsin

Investigations of the functions of melanopsin-mediated visual information in the image-forming vision of humans have inevitable limitations. One way is to gain insight from blind participants with intact melanopsin-related functions.^30^ This is the most straightforward way of separating the visual information received by melanopsin from that received by classical photoreceptors, but it has its disadvantage: from experimental data for such individuals, it is only indirectly possible to infer how melanopsin interacts with other visual signals in sighted individuals. Another useful method is to use metameric, typically melanopsin-isolating, stimuli.^11^^,^^31^^,^^32^^,^^33^ Nevertheless, this method is designed to change the amount of stimulation of one photoreceptor type while keeping other photoreceptors at a constant activation level by metameric lights of suprathreshold intensity. Thus, it is difficult to examine the effect of melanopsin in darkness through this method.

In contrast, the present study applied light stimulation confined within the blind spot, while other regions of the visual field remained untouched; therefore, it provides a rare opportunity to manipulate melanopsin activation without stimulating any other classical photoreceptors. Our findings on the detectability of light in darkness, which would be unattainable by the two methods described previously, indicate that melanopsin may be involved in the functioning of the image-forming pathway under bright illumination conditions where cones predominantly work^2^ and in dark environments where rods mainly operate.

### Involvement of melanopsin in light detection near the absolute threshold

There are two factors in light detection: selecting a potential signal and distinguishing it from noise that inevitably occurs at a certain baseline level. As a model of biological noise generation in addition to external photon noise, there is a hypothesis that the internal noise is directly generated by the activities of rods.^34^ However, because this alone does not adequately explain the magnitude of the noise that limits the detection performance,^35^^,^^36^ downstream activities of neurons, such as bipolar and amacrine cells, have also been proposed.^37^ We argue that the light detection near the absolute threshold is apparently weakened when the blind-spot illumination forces the system involving melanopsin to “sense erroneously” an intensity increase within the entire retina. This means that the noise level estimated by this system, apart from the noise originating from an image-forming pathway, normally helps optimal light detection near the absolute threshold. Individual rods and their vertical transmissions send localized photometric values, similar to noisy photodiodes. Only a system with access to summary statistics can estimate the noise level, which is crucial for detection decisions. Detectability improves if the noise is estimated as prior information and if the estimated noise is more adaptive to changes in the environment, serving as a baseline in reference to which each decision can be made. Such summary statistics can be theoretically obtained by information integration, at a higher level of visual hierarchy in the brain, from all over a million inputs originating from activations in classical photoreceptors. However, we propose that the system involving melanopsin has a similar effect in parallel using a significantly more straightforward mechanism, the wide arborization and interconnectivity of melanopsin-containing ipRGCs within the retina, which is incessantly bombarded with environmental illumination.

The aforementioned hypothesis is neutral about the specific computation protocol to control light-detection sensitivity and its processing stage within the retinogeniculostriate pathway and beyond. Involvement of ipRGCs themselves in active suppression of light detection is suggested in recent physiological studies, in which the amplitudes and firing rates of the retinal ganglion cells,^38^ as well as the visual responses and narrowband oscillations in the dorsal lateral geniculate nucleus (dLGN)^39^^,^^40^ are modulated with the ambient light level. These modulations are lost in melanopsin knockout mice and evoked by the chemogenetic activation of ipRGCs, suggesting their key role in the illumination-dependent modulations. Among such illumination dependency, some ipRGCs release GABA under low illumination, presumably serving as a modulatory circuit for circadian rhythm entrainment and light reflex,^41^ and this may also help achieve a feedback system within the retina, as suggested by a recent silent-substitution study demonstrating that melanopsin stimulation enhances cone sensitivity^32^ but suppresses rod sensitivity.^33^

### Limitations of the study

Our study has not physiologically recorded neural activation of melanopsin-containing ipRGCs in response to the blue-light illumination of the neural tissue running in the optic disk. Thus, the melanopsin involvement we are claiming is currently our best inference by a process of elimination so far. To overcome this drawback, we are currently building biopsychological methodologies to identify a telltale signature of melanopsin by examining how well its known light-absorbance properties explain the human physiological responses to the light illumination confined within the optic disk.

## STAR★Methods

### Key resources table

**Table:** 

REAGENT or RESOURCE	SOURCE	IDENTIFIER
**Deposited data**		

Raw experimental data	This paper	https://doi.org/10.17605/OSF.IO/7KB3H

**Software and algorithms**		

MATLAB	MathWorks	https://www.mathworks.com/
Psychtoolbox	Brainard^42^	http://psychtoolbox.org/

### Resource availability

#### Lead contact

Further information and requests for resources and reagents should be directed to and will be fulfilled by the lead contact, Marina Saito (marina@sda.nagoya-cu.ac.jp).

#### Materials availability

This study did not generate new unique reagents.

#### Data and code availability

• All the data have been deposited at OSF and are publicly available as of the date of publication. The DOI is listed in the key resources table.
• All original code has been deposited at OSF and are publicly available as of the date of publication. The DOI is listed in the key resources table.
• Any additional information required to reanalyze the data reported in this paper is available from the lead contact upon request.

### Experimental model and study participant details

#### Experimental models

We used the ENLIGHT checklist^43^ to show the details of light in our experimental setup (See Figure S4).

#### Observers

The sample size was determined using *a priori* power analysis with G∗Power based on the effect size of our previous study (*α* = 0.05, *β* = 0.80, *d* = 0.8)^2^. The power analysis yielded *N* = 15 as a sufficiently large sample size for a paired t-test; thus, we recruited participants so that the final sample size became larger than 15. In Experiments 1, 2, and 3, 17 (six females and 11 males; mean age, 21.7 years), 18 (seven females and 11 males; mean age, 22.4 years), and 19 (10 females and nine males; mean age, 24.8 years) healthy Asian adult participated, respectively. However, one observer in Experiment 1 and one observer in Experiment 3 were excluded from data analysis because all of the luminance levels for them turned out to be inadequately set (d’ < 1), and one observer in Experiment 1 and two observers in Experiment 2 were excluded because more than half of the data had to be discarded owing to fixation errors. All observers had normal or corrected-to-normal visual acuity. All observers, except the first author in Experiment 3, were naive to the purpose of the experiments. All statistical significances of Experiment 3 remained unchanged even if we excluded the author’s data from the analysis.

This study conformed to the Declaration of Helsinki guidelines and was approved by the Ethics Committee of the Graduate School of Humanities and Sociology at the University of Tokyo (utps-19003). The experiments were conducted in accordance with the approved guidelines. Informed consent was obtained from each observer before the experiment.

### Method details

#### Apparatus

The experiments were conducted in a dark room. All stimuli were displayed on an LCD monitor (VIEWPixx/3D Lite; VPixx Technologies; 1.5 arcmin/pixel, 49.0 deg × 31.7 deg) with a refresh rate of 120 Hz, under the control of a computer (Apple Mac Pro). We used the MATLAB (MathWorks) programming environment, with the Psychophysics Toolbox^42^ and Eyelink Toolbox for programming. Each observer’s head was constrained using a chin rest. The viewing distance was set to 52 cm. The left eye was completely occluded with an opaque patch. During each block, the pupil diameter and gaze position of the right eye were constantly recorded using an eye tracker (SR Research Eyelink II) at a sampling rate of 250 Hz.

#### Stimuli

In Experiments 1 and 3, we presented an achromatic test stimulus, which we called an “arc” (0.0942–0.106 cd/m^2^), on a dark background (0.0896 cd/m^2^). In Experiment 2, the arc (41.16–43.28 cd/m^2^) was presented on a bright gray background (40.86 cd/m^2^. See Figure S2 for spectral components), for which α-opic radiance [L, M, S, rod, melanopsin (W･sr^−1^･m^−2^)] = [0.0695, 0.0643, 0.0356, 0.0848, 0.0823] (CIE Toolbox^44^). The arc was a semicircle covering the perifovea (5–10 deg eccentricity) of the left hemifield. Prior to the blocks of blind-spot illumination, the arc’s luminance was optimized for each observer so that the luminance levels would lie near their absolute (Experiments 1 and 3) or contrast (Experiment 2) thresholds.

In half of the trials of Experiments 1 and 2, 90% of the blind spot area was illuminated by blue light, which we called a “BS stimulus” (2.71 cd/m^2^, CIE [x, y] = [0.143, 0.585], α-opic radiance [L, M, S, rod, melanopsin] = [0.0059, 0.0097, 0.0251, 0.0233, 0.0282]). Its spectral distribution was restricted to a short-wavelength range using a band-pass filter (Fuji Film BPB-45; wavelength peak, 462 nm; full width at half maximum, 19 nm). The conditions in which the BS stimulus was present and absent were called the “Blue Illumination condition” and “No Illumination condition,” respectively. Throughout each trial, the BS stimulus was surrounded by a red annulus (11.14 cd/m^2^, CIE [x, y] = [0.681, 0.303], α-opic radiance [L, M, S, rod, melanopsin] = [0.0222, 0.0045, 0.0001, 0.0008, 0.0005]) that was equivalent to the BS stimulus in area (width: 0.75–0.8 deg). Because the red annulus covered the blind-spot border, the observer always perceived a perceptually filled red oval, regardless of the presence or absence of the BS stimulus.

In Experiment 3, we added a red BS stimulus (2.79 cd/m^2^, CIE [x, y] = [0.698, 0.301], α-opic radiance [L, M, S, rod, melanopsin] = [0.0056, 0.0010, 0.0000, 0.0001, 0.0000]) of the same luminance as the blue BS stimulus and with the spectral distribution restricted to a long-wavelength range using a high-pass filter (Fuji Film SC-54; wavelength peak, 634 nm; full width at half maximum, 24 nm).

#### Procedures

Initially, the blind spot of each observer’s right eye was mapped using the same method as in a previous study.^2^ To the right of a central fixation point, we presented an oval. Each observer adjusted its location and size using a computer mouse and keys. After the oval was turned off, a small disk (0.15 deg in diameter) appeared nearby, to which a speeded keypress was requested. Fifty-six disks (7 distances from the oval border × 8 radial directions from the oval center) were presented in random order. The perimetry was repeated at least eight times. The position corresponding to a 50% detection rate was determined in each radial direction, and adjacent points were connected by straight lines to create a polygon. The polygon’s inscribed oval was determined geometrically and further shrunk to 90% in size to avoid unintended misalignment, and the final outcome was defined as the BS stimulus for each observer. In Experiment 3, the smallest size of the BS stimulus in Experiment 1 was used for all observers to minimize the concern of light leakage.

Light detectability was measured using a two-interval forced-choice (2IFC) task for the detection of the arc (Figure 1), in which either the first or second interval contained an arc with 50% probability. A central fixation point and a red annulus remained on the monitor throughout each block. Regardless of the background luminance, the blind-spot region was consistently dark with zero video output unless the BS stimulus was turned on. In the Blue Illumination condition, each trial began at the onset of the BS stimulus. Subsequently, 650 ms later, the “first interval” for the 2IFC was inserted for 50 ms, and the BS stimulus was turned off at the end of it. After a 150-ms blank, the BS stimulus was again presented for 650 ms prior to the “second interval” inserted for 50 ms, and the BS stimulus was turned off at its end. Each “interval” was preceded by a continuous beep for 600 ms. A third beep was delivered 800 ms after the second interval in Experiments 1 and 2 and 700 ms after the second interval in Experiment 3. Then, the observer was instructed to report whether the arc appeared after the first or second beep by pressing one of two computer keys. The inter-trial interval was randomly selected in the range of 2.5 ± 0.5 s. In the No Illumination condition, the procedure was the same as above, but the BS stimulus was never presented and the blind-spot region remained dark (0.0896 cd/m^2^). In the Blue Preillumination condition of Experiment 3, the procedure was the same as that of the Blue Illumination condition, except that the BS stimulus was turned off 50 ms before each “interval” started. Hence the blind-spot region was dark during each “interval.” Experiment 3 also included the “Red Illumination” and “Red Preillumination” conditions, which were the red counterparts of the abovementioned illumination conditions with blue.

In each of Experiments 1 and 2, 720 trials were performed in 10 blocks. In Experiment 3, 1584 trials were performed in 22 blocks. Within each block, the No Illumination and Blue Illumination (and also Blue Preillumination in Experiment 3) conditions were tested in a random order for the same number of trials. Additionally, in Experiment 3, the blue and red conditions were conducted in alternate blocks, and which color was used first was counterbalanced across observers. Between blocks, each observer took a 5–6 min break in a dimly lit room.

#### Individual optimizations of luminance

Prior to the blocks of blind-spot illumination, a practice block and a few luminance optimization blocks were run only under the No Illumination condition. The practice block was the same as the other blocks, except that feedback about each response error was provided. In the luminance optimization block, the observer initially performed the detection task at three luminance levels (0.0985 cd/m^2^, 0.0994 cd/m^2^, and 0.100 cd/m^2^), and d’ was calculated for each luminance level based on 50 trials for each. We selected the luminance at which the observer’s d’ was approximately 1.1 and two luminance levels neighboring it (i.e., the previous entry and the next entry on the color look-up table), collected >50 trials for each of the three levels, and recalculated d’ at each. This process was iterated until at least one of the three luminance levels yielded d’ within 1.1–1.5.

In each experiment, there were three luminance levels customized as such for each observer, and we confirmed that the effect of the illumination was not dependent on the luminance levels by performing an ANOVA (see Figure S3), which yielded no significant interaction between blind-spot illumination (presence or absence) and luminance level as within-observer factors. The absence of interaction allowed us to calculate an aggregated d’ for which the interval containing an arc at any of the three luminance levels was simply considered the signal-present interval whereas the interval containing no arc was considered the signal-absent interval. Therefore, the results section consistently shows the aggregated d’ for clarity, and individual d’ values calculated for each luminance level for each observer are shown in Figure S3.

### Quantification and statistical analysis

#### Behavioral data

All the analysis and the statistical test were conducted by using MATLAB.

Detection performance (d’) was calculated from the following equation:^45^

d’ = { z(response 1st/trials in which the test arc appeared in the first interval) - z(response 1st/trials in which the test arc appeared in the second interval) }/√2.

In all the experiments, we performed a paired t-test (α = 0.05). Additionally, we conducted a two-way repeated-measures analysis of variance (ANOVA) in Experiment 3 (α = 0.05). ^∗∗∗^*p* < 0.001. ^∗^*p* < 0.05. “n.s.” indicates not significant in figures.

#### Pupil data

Trials that contained eye blink and/or fixation errors (<1 deg) were excluded from the offline analysis. The pupil diameter in each trial was normalized as a fraction of the baseline diameter averaged over the 500-ms period just before the beginning of each trial.
