# Supplementary material for: Illumination by short-wavelength light inside the blind spot decreases light detectability
Source: iScience. 2024 Jul 30;27(9):110612. doi: 10.1016/j.isci.2024.110612 (PMC11363485; doi:10.1016/j.isci.2024.110612)
Supplement: Document S1. Figures S1–S4 [file mmc1.pdf]

iScience, Volume *27*

Supplemental information

Illumination by short-wavelength light inside the blind spot decreases light detectability

Marina Saito, Kentaro Miyamoto, and Ikuya Murakami

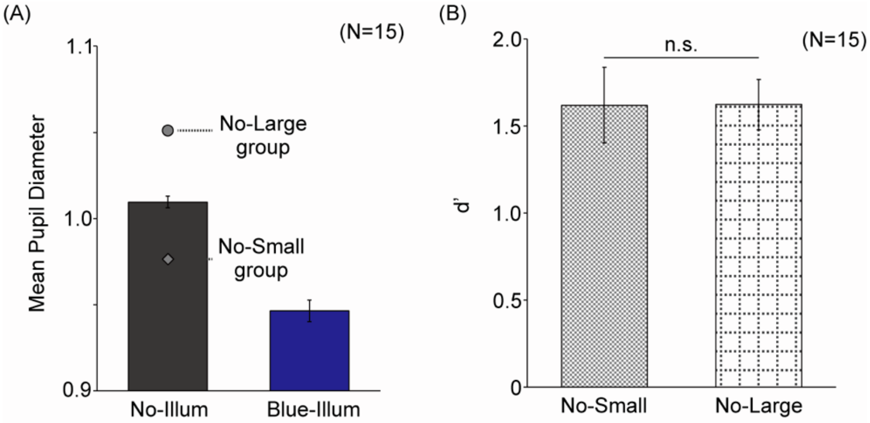

Figure S1: Additional analysis of Experiment 1 using the size of the pupil, Related to Figure 4.
In Experiment 3, we confirmed that differences in retinal illuminance between conditions due to pupil constriction did not affect the detectability. Here, we show the results of the same analysis for the data of Experiment 1.

(A) The mean ± 1 SEM pupil diameter averaged between the first and second intervals. The circles and the diamonds indicate the mean pupil diameters for the No-Large and No-Small groups, respectively, classified according to the protocol described in the main text. As in Experiment 3, the pupil constriction was stronger in the Blue Illumination condition than in the No Illumination condition (t_{14} = 7.03, p = 5.97 × 10^{-6} << .001). The pupil diameter change in the Blue Illumination condition was approximately 7.2% on a grand average. As we averaged the pupil size during the first and second intervals, the interobserver averages in the Blue Illumination and No Illumination conditions were 94.65% and 100.96%, respectively, and their difference, 6.31%, was statistically significant (t_{14} = −8.24, p = 9.69 × 10^{-7}<< .001). When we sorted the trials for the No Illumination condition as in Experiment 3, the difference in pupil size between the two “Small” and “Large” datasets was 7.44%, which was not significantly different from the aforementioned difference, 6.31% (t_{14} = −1.51, p = 0.15 > .05, Figure S1A).

(B) Light detectability for each group. The error bars indicate ± 1 SE. “n.s.” indicates not significant.
Contrary to the retinal illuminance account, there was no difference in d’ between these two datasets under the No Illumination condition (t_{14} = −0.016, p = 0.987 > .05), indicating that pupil size has no effect on the difference in visibility of the test stimulus.

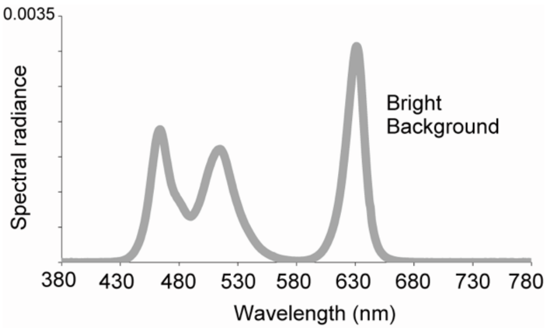

Figure S2: Spectral components of the bright gray background in Experiment 2, Related to Figure 3B.

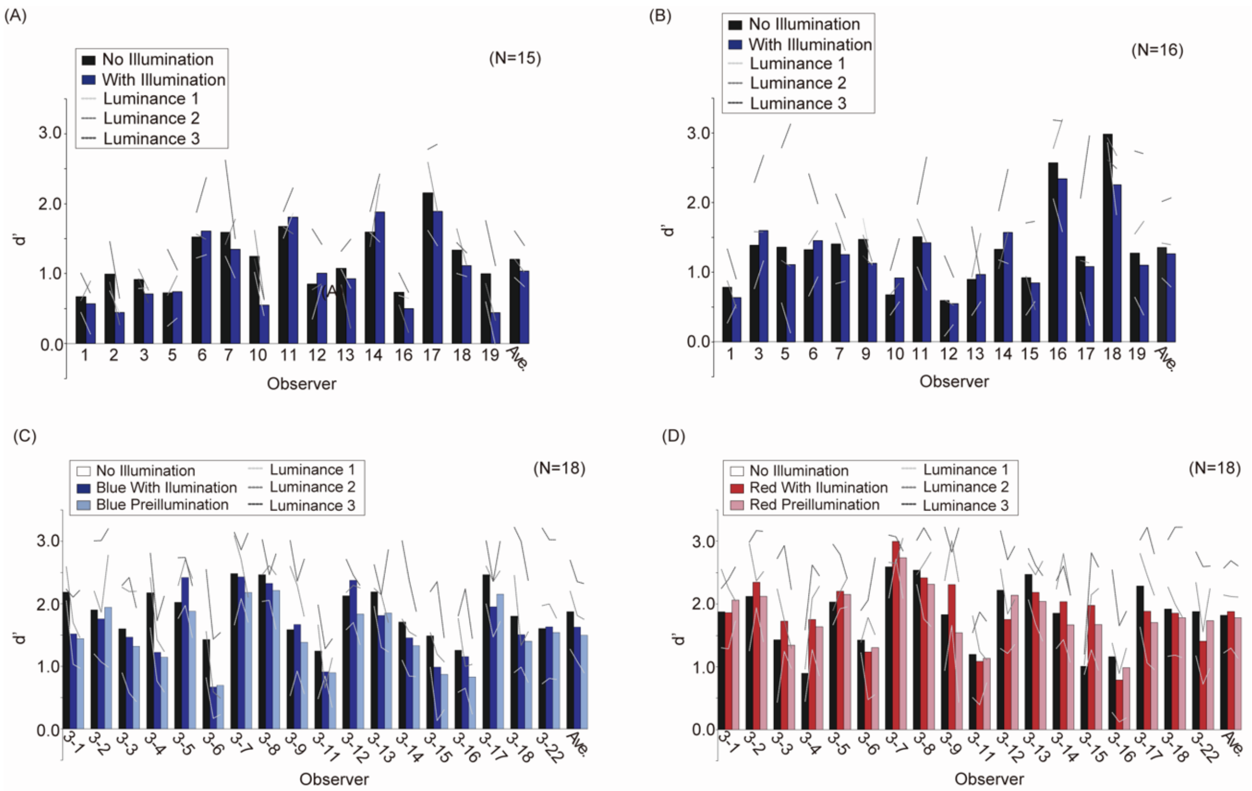

Figure S3: All the data and the analysis of variance (ANOVA) of all experiments, Related to Figures 2 and 3.

To use the value of d' aggregated for the three luminance levels we used in each of the light detection experiments, we had to confirm that the effect of blind-spot illumination was not dependent on the luminance levels. Our statistical analysis allowed us to use aggregated d' for the sake of concise descriptions in all experiments but just as described below, all the statistical results mentioned in the main text also survived when we used the original d' separately calculated for each of the three luminance levels.

(A) Data for all observers included in the data analysis in Experiment 1. To confirm the independency of the luminance, in Experiment 1, we performed a two-way repeated-measures ANOVA for d' with the blind-spot illumination (Blue Illumination or No Illumination condition) and luminance level as within-observer factors. The main effect of blind-spot illumination was marginally significant ($F[1, 14] = 4.50$, $p = 0.052 < .10$). We also found a significant main effect of luminance level, as expected ($F[2, 28] = 25.20$, $p = 5.50 \times 10^{-7} << .001$), and significant differences between all pairs of the three levels were also confirmed by Tukey–Kramer's method ($p < .05$). However, we found no significant interaction between blind-spot illumination and luminance level ($F[2, 28] = 0.23$, $p = 0.80 > .05$), indicating that the effect of blind-spot illumination is not luminance-level-dependent within the examined range.

(B) Data for all observers included in the data analysis in Experiment 2. All observers except 15 also participated in Experiment 1. The ANOVA for the data of Experiment 2 revealed no significant main effect of blind-spot illumination (Blue Illumination or No Illumination condition) ($F[1, 15] = 0.29$, $p = 0.60 > .05$). Additionally, although the main effect of luminance level was significant ($F[2, 30] = 36.12$, $p = 1.03 \times 10^{-8} << .001$), the interaction between blind-spot illumination and luminance level was not ($F[2, 30] = 0.71$, $p = 0.50 > .05$), implying that the blind-spot illumination has no effect at any luminance level.

(C) Data for all observers included in the data analysis in the blue conditions of Experiment 3.

(D) Data for all observers included in the data analysis in the red conditions of Experiment 3. In Experiment 3, we performed a three-way repeated-measures ANOVA with color (blue or red), blind-spot illumination (Blue/Red Illumination, Blue/Red Preillumination, or No Illumination condition), and luminance level as within-observer factors. The main effect of color ($F[1, 17] = 15.93$, $p = 0.00095 < .001$), luminance level ($F[2, 34] = 197.02$, $p = 1.99 \times 10^{-19} << .001$), and blind-spot illumination ($F[2, 34] = 12.87$, $p = 6.90 \times 10^{-5} << .001$) were significant. However, there was no significant interaction between color and luminance level ($F[2, 34] = 0.27$, $p = 0.76 > .05$), and there was no significant interaction between blind-spot illumination and luminance level ($F[4, 68] = 0.90$, $p = 0.47 > .05$), indicating that the effect of blind-spot illumination is not luminance-level-dependent in any sense. However, the interaction between color and blind-spot illumination was highly significant ($F[2, 34] = 6.05$, $p = 0.0056 < .01$), as evident in Figure 3b. Finally, there was no significant three-way interaction among color, luminance level, and blind-spot illumination ($F[4, 68] = 0.33$, $p = 0.85 > .05$).

Note: This document is best viewed and completed with Adobe Acrobat Reader.

ENLIGHT

Release 1.0.2, 16 October 2023

ENLIGHT Checklist

Below is the **ENLIGHT Checklist** for reporting ocular light exposures in human laboratory-based studies. We will strongly encourage that this checklist be used in conjunction with the **ENLIGHT Explanation & Elaboration (E&E) document**. This checklist is intended both to help authors, reviewers, and editors in evaluating the completeness of reporting in submitted studies, and for documentation of studies after publication. In the location column, please indicate the page, figure, or table number where the item or description can be found. If an item is not available, please select "**Not available**". If you consider an item not to be applicable in your specific study design after consulting the guidelines, please select "**Not applicable**". Items which do not have the option to select "Not applicable" were rated by experts as applicable for all studies, regardless of context. If you are unable to provide the information, please select "Not available".

The **ENLIGHT Checklist** (this document) and the **ENLIGHT E&E document** are released under the CC-BY-NC-ND License. For more information, please visit http://enlight-statement.org/.

General Information

Author names: Marina Saito, Kentaro Miyamoto, Ikuya Murakami

Title of manuscript: Illumination by short-wavelength light inside the blind spot decreases light detecability

Date: Jun 21th, 2024

A. Study Characteristics

A.1. Protocol-level characteristics

	Location (page, figure, table number)	Not available	Not applicable
Description of experimental setting	page17	☐	
Timeline of experiment (including timing and duration of light)	Figure1, page18	☐	
Pre-laboratory sleep-wake/rest-activity behaviour		☐	☑
Pre-laboratory light exposure	page18	☐	☐
Immediate prior light exposure (in laboratory)		☐	☑

A.2. Measurement-level characteristics

Measurement plane (e.g., horizontal or vertical)		☑	
Measurement viewpoint and location		☑	
Type, make and manufacturer of the measurement instrument		☑	
Calibration status of the instrument		☐	☑

A.3. Participant-level characteristics

Ocular health and functioning	page16	☐	
Pupil size and/or dilation	Figure4, page9	☐	☐
Relative time (e.g. to circadian phase or sleep)		☐	☑

1

ENLIGHT

Release 1.0.2, 16 October 2023

B. Light characteristics

B.1. Light source type(s). Please select all that are relevant.

Room illumination (overhead or other)	Emissive surfaces including displays (incl. light therapy devices)	Wearable light emitting glasses	Ganzfeld exposure	Other:
☐	☐	☐	☐	☐

Polychromatic light	Monochromatic or narrowband light
☐	☑

	Location (page, figure, table number)	Not available	Not applicable
Type, make and manufacturer of the light source	page17	☐	
Use of wearable filtering apparatus (e.g., blue-blocking glasses)		☐	☑

B.2. Light level characteristics

Illuminance (lux) and/or luminance (cd/m^2)	page17	☐	
Spectral irradiance and/or radiance distribution	Figure3B	☐	☐
α−opic irradiance and/or radiance (including melanopic)	page17	☐	☐
α−opic equivalent daylight illuminance and/or luminance (EDI/EDL, including melanopic)		☐	☑

NOTE: Luminance and radiance metrics (as opposed to illuminance and irradiance) are mainly relevant for emissive surfaces.

B.3. Colour characteristics

Peak wavelength and bandwidth	page17	☐	☐
Colour appearance quantities (any)		☐	☑
Colour rendering metrics (any)		☐	☑

NOTE: Peak wavelength and bandwidth are most relevant for monochromatic or narrowband light sources.

B.4. Temporal and spatial characteristics

Location of stimulus and viewing distance	page17	☐	
Temporal pattern (including flash frequency and waveform)		☐	☑
Relative or absolute size of the stimulus	page17	☐	☐

Reset form to default values | Print as PDF

2

Figure S4: The ENLIGHT Checklist, Related to STAR Methods.
